# *GNAS* mutation is an unusual cause of primary adrenal insufficiency: a case report

**DOI:** 10.1186/s12887-022-03517-6

**Published:** 2022-08-04

**Authors:** Yajie Tong, Dongmei Yue, Ying Xin, Dan Zhang

**Affiliations:** Department of Pediatrics, Shengjing Hospital of China Medical University, Shenyang, Liaoning 110004 P. R. China

**Keywords:** *GNAS* mutation, Primary adrenal insufficiency, Congenital hypothyroidism, Pseudohypoparathyroidism, Case report

## Abstract

**Background:**

Primary adrenal insufficiency in children has non-specific and extensive clinical features, so the diagnosis of its etiology is complex and challenging. Although congenital adrenal hyperplasia is the most common cause, more and more other genetic causes have been identified. *GNAS* mutation is easily overlooked as a rare cause of primary adrenal insufficiency. Here we firstly report a neonatal case of primary adrenal insufficiency caused by *GNAS* mutation.

**Case presentation:**

A boy was diagnosed with congenital hypothyroidism 10 days *post-partum* and treated immediately. He also had persistent hyperkalaemia and hyponatraemia with elevated adrenocorticotropic hormone. At 70 days after birth, he was transferred to our hospital on suspicion of congenital adrenal hyperplasia. Physical examination found no other abnormalities except for growth retardation. Laboratory examination revealed increased aldosterone and normal cortisol, 17-hydroxyprogesterone, and androstenedione levels. Abnormally elevated parathyroid hormone was accompanied by normal blood calcium. Genetic assessment found a de novo, heterozygous c.432 + 1G > A variant in *GNAS*.

**Conclusions:**

We report this case to highlight that *GNAS* mutation is an unusual cause of primary adrenal insufficiency. The combination of primary hypothyroidism and /or pseudohypoparathyroidism will provide diagnostic clues to this condition.

## Background

Primary adrenal insufficiency (PAI) is a rare and potentially life-threatening disease in children. Due to the non-specific and extensive clinical features, the etiological diagnosis of PAI becomes challenging. PAI in childhood is mostly caused by monogenic diseases, and congenital adrenal hyperplasia (CAH) is the most common etiology [1–3]. However, other non-CAH genetic etiologies have been gradually established, such as *NR5A1*/*SF-1*, *NROB1*/*DAX-1*, *ABCD1* and *PEX1*. The *GNAS* gene encodes the alpha-subunit of the G protein (Gsα) which mediates the signalling of numerous peptide hormones such as parathyroid hormone (PTH), thyroid stimulating hormone (TSH), growth hormone–releasing hormone (GHRH), adrenocorticotropic hormone (ACTH), and gonadotrophins. A maternally derived *GNAS* mutation causes the Albright hereditary osteodystrophy (AHO) phenotype (short stature, obesity, round face, subcutaneous ossifications, brachydactyly, mental deficits, and intrauterine growth restriction), with multiple hormone resistance (MHR). However, paternally derived mutations may lead to only AHO without hormone disorders [4]. A large number of reports have confirmed that *GNAS* mutations can cause pseudohypoparathyroidism (PHP), congenital hypothyroidism (CH) and growth hormone deficiency (GHD) due to hormone resistance. However, conclusions about whether *GNAS* mutations cause ACTH resistance and PAI are inconsistent. Here we report for the first time a neonatal case of PAI caused by *GNAS* mutation, indicating that *GNAS* mutation is a potential and unusual cause of PAI.

## Case presentation

The patient from a nonconsanguineous family was a premature boy with a gestational age of 36 w and a birth weight of 1.9 kg. After birth, he was diagnosed with congenital hypothyroidism in a local hospital and treated immediately (L-thyroxine 25 μg/q.d p.o.). Furthermore, hyperkalaemia and hyponatraemia were noted during the same admission and persisted after discharge without treatment. At the same time, serum ACTH, renin, and aldosterone levels were elevated (Table 1). At 70 days *post-partum*, he was transferred to our hospital on suspicion of CAH. On physical examination, he was found to weigh 2.8 kg (<3rd percentile), without hypotension, skin pigmentation, and signs of AHO. His external genital development was normal. Hypothyroidism had been corrected with L-thyroxine treatment. Test results for the presence of thyroid auto-antibodies were negative, and ultrasonography scan showed no goitre. Hyperkalaemia and hyponatraemia were confirmed. Serum calcium and 25-hydroxyvitamin D3, blood glucose, and renal function were normal. On hormonal assessment, elevated ACTH and aldosterone levels and a normal basal cortisol level were detected. The follicle-stimulating hormone (FSH), luteinizing hormone (LH), and testosterone concentrations were matched with ‘mini-puberty’ (Table 1). The 17-hydroxyprogesterone (17-OH-P) and androstenedione levels and adrenal ultrasonography scan were normal. After intravenous infusion of normal saline, serum potassium decreased to slightly higher than normal, and serum sodium rose to normal, such that hydrocortisone was not used. After obtaining the parents’ informed consent, genomic DNA of the baby and his parents was genetically tested.

**Table 1:** Laboratory evaluations at diagnosis and follow-up visits

Age	Investigation	Result	Normal Range
10 days	TSH	58.43 uIU/ml	0.3–5.7
	FT_4_	6.8 pmol/L	10–28
20 days	serum potassium	6.7 mmol/L	3.5–5.5
	Serum sodium	131 mmol/L	135–145
	ACTH (8:00)	20.56 pmol/L	1.6–13.9
	Renin	49.5 pg/mL	4–24
	aldosterone	959.77 pg/mL	10–160
70 days	serum potassium	6.13 mmol/L	3.5–5.5
	Serum sodium	127.7 mmol/L	136–145
	Serum calcium	2.26 mmol/L	1.9–2.6
	Serum phosphate	1.94 mmol/L	1.2–1.9
	ACTH (8:00)	111.8 pg/mL	7.2–63.3
	cortisol	10.00 μg/dL	6.02–18.4
	aldosterone	380.1 pg/mL	30–160
	LH	7.20 mIU/mL	
	FSH	3.23 mIU/mL	
	testosterone	2.24 ng/mL	
	17-OH-P	5.42 ng/ mL	3.6–13.7
	androstenedione	1.10 ng/mL	0.6–3.1
6 months	serum potassium	6.62 mmol/L	3.5–5.5
	Serum sodium	129 mmol/L	136–145
	Fasting glucose	5.17 mmol/L	3.9–6.11
	ACTH (8:00)	34.34 pg/mL	7.2–63.3
	cortisol	7.64 μg/dL	6.02–18.4
	aldosterone	478.2 pg/mL	70–300
	LH	0.53 mIU/mL	
	FSH	1.57 mIU/mL	
	testosterone	0.14 ng/mL	
	Serum calcium	2.38 mmol/L	1.9–2.6
	Serum phosphate	1.95 mmol/L	1.2–1.9
	PTH	135.4 pg/mL	12–88
	IGF-1	26.93 ng/mL	15–305

*TSH* thyroid stimulating hormone, *FT*_*4*_ free thyroxine, *ACTH* adrenocorticotropic hormone, *LH* luteinizing hormone, *FSH* follicle-stimulating hormone, *17-OH-P* 17-hydroxyprogesterone, *PTH* parathyroid hormone, *IGF-1* insulin-like growth factor-1

After being discharged from our hospital, the child returned to his local hospital for a follow-up examination. Thyroid function was well controlled, but hyperkalaemia and hyponatraemia persisted. At the age of 6 months, the child came back for re-examination. He weighed only 5.3 kg (<3rd percentile) and had normal blood pressure and neuromotor development. Growth was slow, although euthyroid with levothyroxine, persistent hyperkalaemia, hyponatraemia, and increased aldosterone suggested that there was still cortisol insufficiency. Elevated serum PTH with normal serum calcium and slightly elevated phosphate levels indicated resistance to PTH. Renal function and 25-hydroxyvitamin D3 remained normal. Serum LH, FSH, and testosterone levels were consistent with his age. Serum insulin-like growth factor-1(IGF-1) concentration was at a low value in the normal range (Table 1).

**Fig. 1:**
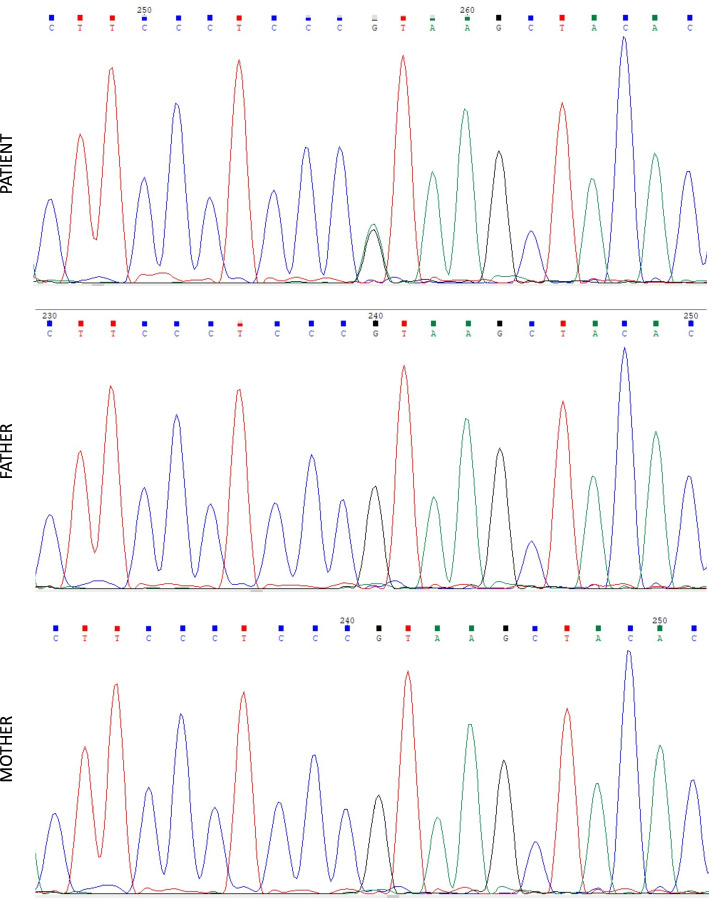
The heterozygous c.432 + 1G > A mutation in the *GNAS* gene was detected in the patient’s lymphocytes, but was absent in the parental blood samples

Next generation sequencing of the patient’s DNA revealed a de novo heterozygous c.432 + 1G > A variant in *GNAS* (Fig. 1). It is a splicing mutation affecting the canonical splice donor site of intron 5 and is likely to result in loss of the donor splice site. According to the ACMG guideline, this variant is considered to be pathogenic. Literature review indicated that this variant had been previously described to be related to AHO [5]. To date, only five other cases with the same mutation have been reported, of which three patients had the mutation in the paternal allele and exhibited features of AHO but had no hormone resistance [5, 6]. The other two patients’ clinical phenotypes and biochemical and endocrine characteristics were not described in detail by the authors [7, 8]. Our case is the first report that this mutation causes MHR. Since only inactivating mutations that occur in the maternal allele can cause MHR, it is speculated that the de novo mutation of our case occurred in the maternal allele.

## Discussion and conclusions

In this report we present a patient with PAI and primary hypothyroidism in the neonatal period, genetic assessment revealed a heterozygous variant in *GNAS* as an underlying defect. *GNAS* is located on chromosome 20q13.2–13.3 and is a complex imprinted gene. Owing to tissue-specific imprinting, in some tissues (such as renal proximal tubule, thyroid, pituitary, and gonads), paternal alleles are not expressed, so loss-of-function mutations of maternal alleles can lead to decompensated Gsα protein inactivation then result in resistance to PTH, TSH, GHRH, and gonadotrophins. However, in most tissues, because of biallelic *GNAS* expression, a heterozygous mutation is not sufficient to significantly interfere with gene function. Paternal mutations can cause AHO, possibly owing to haplo-insufficiency of Gsα in bone tissue [9].

Cases with GNAS mutations have diverse clinical phenotypes, and hormone resistance can be detected at different stages of life with large individual differences [10, 11]. The onset of PTH resistance is usually delayed and may not be discovered until childhood, adolescence, or even adulthood. This latency of PTH resistance may be due to a gradual development of paternal Gsα silencing in the maternally imprinted tissues [12]. Similar to previous cases [13], our patient had normal serum calcium with an inappropriately raised PTH level, excluding secondary hyperparathyroidism caused by renal insufficiency and vitamin D deficiency. This indicates the emergence of PTH resistance, and long-term follow-up is required to monitor the serum calcium level.

Besides PTH, resistance to other hormones may also be seen. Among them, TSH resistance is the most common and usually the first to be discovered. It manifests as an elevated TSH with normal or slightly low FT4, usually in the absence of goitre and anti-thyroid antibodies. Elevated TSH may be detected at neonatal screening and diagnosed as congenital hypothyroidism [14], just like that in this case. It may also be discovered in infancy or even in childhood and adolescence.

Although corticotropin-releasing hormone (CRH) and ACTH function through Gsα receptors, there are inconsistent conclusions about whether GNAS mutation can cause adrenal insufficiency. Ridderskamp et al. [15] believe that PHP patients can be accompanied by ACTH resistance, as they detected hypocortisolism in an adult PHP case. This opinion was supported by three other reports which found elevated ACTH levels or an exaggerated ACTH response to CRH in PHP patients [16–18]. Chaubey et al. [19] also reported a case of GNAS mutation with primary adrenal insufficiency, although the author believes that the adrenal insufficiency was idiopathic. Our patient developed hyperkalaemia, hyponatraemia, and increased ACTH during the neonatal period and grew slowly, even with normal thyroid function control. CAH was once suspected, but the child had no clinical manifestations of elevated androgen; 17-OH-P and androstenedione were normal, aldosterone was increased, and adrenal ultrasonography scan was normal. The diagnosis of classic CAH, congenital adrenal dysplasia, and adrenal haemorrhage was not supported. Combined with the results of genetic testing, the condition was attributed to adrenal insufficiency caused by ACTH resistance. However, some other reports indicate normal adrenal responsiveness to ACTH/CRH in patients with PHP [20, 21]. It is speculated that the absence of ACTH resistance may be related to the biallelic expression of the GNAS gene in the adrenal gland. Previous studies on pituitary, thyroid, and gonadal samples have revealed significant inter-individual variation with respect to the degree of paternal Gs expression [22, 23]. If there is a similar situation in the adrenal gland, the variable degree of paternal *Gs* silencing in the adrenal gland may be a possible explanation why ACTH resistance is less frequently encountered. In addition to the residual Gsα function and imprinting mechanism, other modification factors, such as environmental factors and cofactors of the cAMP coupling pathway, may also affect the clinical phenotype of patients with *GNAS* mutations. Further studies are required to explain the individual phenotypic variability among these patients.

About two-thirds of PHP patients are reported to develop GH deficiency, secondary to GHRH resistance [11, 21, 22, 24]. GH deficiency and the premature fusion of the epiphyses result in the patient’s short stature. Studies have shown that rhGH replacement therapy before puberty can increase the growth rate and maximally improve the final adult height [9]. Our case had growth retardation, and the IGF-1 level of the patient was at a low value in the normal range. The possibility of combined GHRH resistance could not be excluded. After adrenal insufficiency has been corrected, close monitoring of growth velocity and IGF-1 level is necessary to further evaluate the GH level.

As hypogonadism can also be seen in PHP due to gonadotropin resistance [11], we also need to pay attention to delayed or incomplete sexual maturation during follow-up in our patient.

In conclusion, the causes of PAI are complex and diverse. Due to considerable overlap in clinical and biochemical characteristics of PAIs of different etiology, genetic detection is often required for accurate cause diagnosis. The specific genetic diagnosis of PAI is not only conducive to early diagnosis and reasonable treatment, but also extremely valuable for predicting prognosis and potential comorbidities. Our case suffered from PAI and primary hypothyroidism during the neonatal period, genetic testing confirmed that *GNAS* mutation is the cause. Sharing this case can remind pediatricians not to ignore that *GNAS* mutation is an unusual cause of PAI. The combination of primary hypothyroidism and /or PHP will provide clues to the etiological diagnosis of PAI.

## Data Availability

The gene sequencing data of *GNAS* is stored in NCBI Sequence Read Archive (SRA) (accession number: SRR20281056).

## Abbreviations

PAI: Primary adrenal insufficiency
CAH: Congenital adrenal hyperplasia
Gsα: Alpha-subunit of the G protein
PTH: Parathyroid hormone
TSH: Thyroid stimulating hormone
GHRH: Growth hormone–releasing hormone
ACTH: Adrenocorticotropic hormone
AHO: Albright hereditary osteodystrophy
MHR: Multiple hormone resistance
PHP: Pseudohypoparathyroidism
CH: Congenital hypothyroidism
GHD: Growth hormone deficiency
FSH: Follicle-stimulating hormone
LH: Luteinizing hormone
17-OH-P: 17-hydroxyprogesterone
IGF-1: Insulin-like growth factor-1
CRH: Corticotropin-releasing hormone

## References

[1] Perry R, Kecha O, Paquette J, Huot C, Van Vliet G, Deal C (2005). Primary adrenal insufficiency in children: twenty years experience at the Sainte-Justine Hospital, Montreal. J Clin Endocrinol Metab.

[2] Wijaya M, Huamei M, Jun Z, Du M, Li Y, Chen Q, Chen H, Song G (2019). Etiology of primary adrenal insufficiency in children: a 29-year single-center experience. J Pediatr Endocrinol Metab.

[3] Buonocore F, Maharaj A, Qamar Y, Koehler K, Suntharalingham JP, Chan LF, Ferraz-de-Souza B, Hughes CR, Lin L, Prasad R (2021). Genetic analysis of pediatric primary adrenal insufficiency of unknown etiology: 25 years’ experience in the UK. J Endocr Soc.

[4] Mantovani G, Spada A, Elli FM (2016). Pseudohypoparathyroidism and Gsɑ-cAMP-linked disorders: current view and open issues. Nat Rev Endocrinol.

[5] Wilson LC, Oude Luttikhuis ME, Clayton PT, Fraser WD, Trembath RC (1994). Parental origin of Gs alpha gene mutations in Albright’s hereditary osteodystrophy. J Med Genet.

[6] Rickard SJ, Wilson LC (2003). Analysis of GNAS1 and overlapping transcripts identifies the parental origin of mutations in patients with sporadic Albright hereditary osteodystrophy and reveals a model system in which to observe the effects of splicing mutations on translated and untranslated messenger RNA. Am J Hum Genet.

[7] Tokita MJ, Nahas S, Briggs B, Malicki DM, Mesirov JP, Reyes IAC, Farnaes L, Levy ML, Kingsmore SF, Dimmock D (2019). Biallelic loss of GNAS in a patient with pediatric medulloblastoma. Cold Spring Harb Mol Case Stud.

[8] Aldred MA, Trembath RC (2000). Activating and inactivating mutations in the human GNAS1 gene. Hum Mutat.

[9] Mantovani G, Ferrante E, Giavoli C, Linglart A, Cappa M, Cisternino M, Maghnie M, Ghizzoni L, de Sanctis L, Lania AG (2010). Recombinant human GH replacement therapy in children with pseudohypoparathyroidism type Ia: first study on the effect on growth. J Clin Endocrinol Metab.

[10] Wémeau JL, Balavoine AS, Ladsous M, Velayoudom-Cephise FL, Vlaeminck-Guillem V (2006). Multihormonal resistance to parathyroid hormone, thyroid stimulating hormone, and other hormonal and neurosensory stimuli in patients with pseudohypoparathyroidism. J Pediatr Endocrinol Metab.

[11] Mantovani G, Spada A (2006). Resistance to growth hormone releasing hormone and gonadotropins in Albright’s hereditary osteodystrophy. J Pediatr Endocrinol Metab.

[12] Usardi A, Mamoune A, Nattes E, Carel JC, Rothenbuhler A, Linglart A (2017). Progressive development of PTH resistance in patients with inactivating mutations on the maternal allele of GNAS. J Clin Endocrinol Metab.

[13] Tamada Y, Kanda S, Suzuki H, Tajima T, Nishiyama T (2008). A pseudohypoparathyroidism type Ia patient with normocalcemia. Endocr J.

[14] Pinsker JE, Rogers W, McLean S, Schaefer FV, Fenton C (2006). Pseudohypoparathyroidism type 1a with congenital hypothyroidism. J Pediatr Endocrinol Metab.

[15] Ridderskamp P, Schlaghecke R (1990). Pseudohypoparathyreoidismus und Nebennierenrindeninsuffizienz. Ein Fall von Multipler Endokrinopathie bei peripherer Hormonresistenz [Pseudohypoparathyroidism and adrenal cortex insufficiency. A case of multiple endocrinopathy due to peripheral hormone resistance]. Klin Wochenschr.

[16] Coutant R, Carel JC, Mathivon L, Boisson Lesage C, Renier D, Garabédian M, Chaussain JL (1997). Hypothyroïdie compensée révélant une pseudohypoparathyroïdie en l’absence d’hypocalcémie et d'hyperphosphorémie [Primary hypothyroidism revealing pseudohypoparathyroidism without hypocalcemia and hyperphosphoremia]. Arch Pediatr.

[17] Tsai KS, Chang CC, Wu DJ, Huang TS, Tsai IH, Chen FW (1989). Deficient erythrocyte membrane Gs alpha activity and resistance to trophic hormones of multiple endocrine organs in two cases of pseudohypoparathyroidism. Taiwan Yi Xue Hui Za Zhi.

[18] Long XD, Xiong J, Mo ZH, Dong CS, Jin P (2018). Identification of a novel GNAS mutation in a case of pseudohypoparathyroidism type 1A with normocalcemia. BMC Med Genet.

[19] Chaubey SK, Sangla KS (2014). A sporadic case of pseudohypoparathyroidism type 1 and idiopathic primary adrenal insufficiency associated with a novel mutation in the GNAS1 gene. Endocr Pract.

[20] Downs RW, Levine MA, Drezner MK, Burch WM, Spiegel AM (1983). Deficient adenylate cyclase regulatory protein in renal membranes from a patient with pseudohypoparathyroidism. J Clin Invest.

[21] Mantovani G, Maghnie M, Weber G, De Menis E, Brunelli V, Cappa M, Loli P, Beck-Peccoz P, Spada A (2003). Growth hormone-releasing hormone resistance in pseudohypoparathyroidism type ia: new evidence for imprinting of the Gs alpha gene. J Clin Endocrinol Metab.

[22] Hayward BE, Barlier A, Korbonits M, Grossman AB, Jacquet P, Enjalbert A, Bonthron DT (2001). Imprinting of the G(s)alpha gene GNAS1 in the pathogenesis of acromegaly. J Clin Invest.

[23] Mantovani G, Ballare E, Giammona E, Beck-Peccoz P, Spada A (2002). The gsalpha gene: predominant maternal origin of transcription in human thyroid gland and gonads. J Clin Endocrinol Metab.

[24] Germain-Lee EL, Groman J, Crane JL, Jan de Beur SM, Levine MA (2003). Growth hormone deficiency in pseudohypoparathyroidism type 1a: another manifestation of multihormone resistance. J Clin Endocrinol Metab.

